# The Enno Sander Prize Essay, 1906

**Published:** 1906-11

**Authors:** James Evelyn Pilcher

**Affiliations:** U. S. Army, Secretary of the Association of Military Surgeons of the United States and Editor of the Journal of the Association of Military Surgeons of the United States


### The Enno Sander Prize Essay, 1906.

### The Training of the Medical Officer of the State Forces to Best Qualify Him for Local Service and for Mobilization with National Troops.

By MAJOR JAMES EVELYN PILCHER, U. S. Army.
Secretary of the Association of Military Surgeons of the United States and Editor of the Journal of the Association of Military Surgeons of the United States.

*(Author's Abstract.)*

THE early days of every war, requiring the aggregation of large bodies of men, have been fraught with disease and disaster to the troops which have been gathered. The impression has prevailed that any man can become a soldier after a few days instruction. The result of this notion has been in the highest degree disastrous to the nations where the idea existed. France, Germany and England have alike suffered from this cause. In the last two wars in which the United States has been involved the condition has been particularly conspicuous. Much criticism was turned upon General McClellan because of his failure to move out upon the enemy in the Civil War, but McClellan recognized the fact that an army cannot be made in a day, and an inefficient instrument, no matter what its size, may be brought to wreck by its premature application. The success which crowned the arms of the North in that bloody conflict came only after an army had been created by long experience and active operations.

In the Spanish-American war the conditions might have been more advantageous had the state forces, of which the troops were largely composed, been uniformly trained, so as to combine easily and readily into a national army. It is not within the limits of this essay to specify the needs of a volunteer army nor to criticise or comment upon the errors of other days, but it is hoped that it may open up a discussion which may result, in case of future hostilities, in a nearer approach to harmony among the units of which our forces are composed than has hitherto existed.

The subject of the training of the medical officer of the state forces to best qualify him for local service and for mobilization with national troops is a topic, the importance of which in a nation like the United States of America, cannot be overestimated. Relying as we do upon the state troops as a supplement to the small national army supported by the country, it is essential that the organization of both bodies be so harmonized that service together may occur without the slightest friction or difficulty. This end is sought by the use, in both bodies, of the same drill books, so that in matters of drill, these two branches of the military service would seem to be practically uniform. In other respects, however, this would not naturally occur, as, in the absence of definite instructions, individuality is sure to have its influence.

The writer was greatly impressed by this fact when at the opening of the Spanish-American War he was detached from the regular service and assigned to duty with a volunteer force, which afterward formed the basis of a corps of the volunteer army and itself became a division. The medical conditions which prevailed upon his arrival at the volunteer command were what might have been expected. There were a sufficient number of devoted and successful medical men. Some of the regiments had a hospital organisation; some had tents fitted up for hospital purposes; some had a definite sick call; all had hospital stewards, and some had a nominal hospital corps detachment. There was, however, no general hospital organization, and no hospital corps detachment aside from the privates detailed from the line in some of the regiments. There was little if any attempt to properly record the case histories of the sick men, and the duty and authority of the medical man in the supervision of the sanitation of the camp was not understood. The idea of property responsibility was wholly absent and the duty of the medical officer to the government as a protector from future improper claims for pension was not appreciated.

Early efforts were made to render the service uniform. Not much time was given to directing the professional care of the sick, since it was believed that the medical officers were all well qualified physicians and entirely competent to direct the treatment of the sick of their organizations. But the teaching inaugurated was directed especially to those features in which the medical officers were particularly deficient.

To this fact is due perhaps the criticism made in a recent medical journal by a distinguished physician, who was surgeon of one of the regiments,—that the main object of the administrative medical officer during his service appeared to be the development of records and papers, he apparently not appreciating the fact that his medical qualifications were not considered to demand instruction.

It must be admitted, however, that at the time of the outbreak of the Spanish-American War definite plans for the expansion of the Medical Department had not been made. Many of the medical officers of the regular service had given much thought to the subject and a number of them had written to a greater or less extent upon the topic. Definite arrangements, however, for expansion appear to have been entirely lacking and each regimental, division, and corps surgeon was allowed to pursue his own sweet way. The result was a confusion and a lack of uniformity which was pronounced in the extreme.

As the officer detailed by the Chief Surgeon of General Shafter's force of the regular army at Tampa to inspect the sanitary features of the organisation composing it, the writer examined most of the hospitals comprising its medical department and found no two alike. Most of them were admirable, all of them were ably organized, but there was entire lack of that uniformity which would render consolidation readily and easily accomplished. This was due to the fact that in 1898 there was no definite regimental, brigade, or division hospital organization prescribed in the American service.

It will be seen then from these observations that at the opening of the Spanish-American War there were factors involving both the regular service and the volunteer forces which rendered consolidation and mobilization impossible without material changes in both. That they ultimately came together after a not inconsiderable amount of friction, by which the irregularities were rubbed off and a certain amount of dove-tailing was allowable is due to the high character of the men comprising the personnel rather than to the conditions existing in either branch.

The lesson to be derived from this experience is that in order to admit of prompt assimilation the militia must be trained along lines uniform with those of the national service.

The position of a National Guard medical officer is fraught with responsibility and cannot be taken too seriously. The National Guard is a military body with service extending not only in the temporary summer encampments but throughout the entire year and with duties of the highest importance. It is much more than an opportunity to wear gold lace in winter and go on a week's picnic in summer. It is preparation for grim war which may come at any moment, as well to the peaceful guardsman as to the professional regular. So long as a man connects himself with the citizen soldiery, so long he carries the burden of an obligation to prepare himself for the best possible performance of the duties which he is or may be called upon to perform. He must not feel that with a uniform, his pocket medicine and instrument cases, and a saddle equipment for his horse, he has fitted himself for the work of the military surgeon.

### THE SPECIALTY OF MILITARY MEDICINE.

Military medicine and surgery is a specialty, not difficult of acquisition, it is true, but nevertheless a specialty, proper familiarity with which is essential to the adequate performance of the medical attendance upon a body of military men, be it a separate company, a battalion, a regiment, a brigade, a division or a corps.

To qualify in the specialty of military medicine and surgery, the newly appointed medical officer must become familiar,—in addition to the features of ordinary civilian practice,—with,

I. The special professional knowledge included under—
  1. Military medicine.
  2. Military surgery.
  3. Military hygiene.

II. Medico-military administration, including—
  1. The selection of men.
  2. Definite organisation of work.
  3. The command of men.
     (a) The Hospital Corps.
     (b) The sick.
  4. Camp location and organisation.
  5. Field hospital construction and composition.
  6. The instruction and drill of the sanitary soldier.
  7. "Paper work."

III. The evolution and maintenance of a medico-military esprit de corps.
  1. National patriotism and state loyalty.
  2. Contact with men and literature.

It is evident that the work of the military medical officer is of three kinds, medical, military, and patriotic.

I. THE SPECIAL PROFESSIONAL KNOWLEDGE.

The medical work naturally falls into two classes, therapeutic and prophylactic.

*Therapeusis.*—The therapeutic work of the military medical officer differs but little from that of the civilian practitioner in its simpler forms, and any practitioner who has mastered the curriculum of one of the numerous high grade medical schools, which are so abundant in the United States, should be competent to conduct this phase of military medical work without especial instruction.

A military dispensary, however, must necessarily be mobile and consequently exclude elaborate preparations and confine itself to one of several agents, each of which may accomplish the same purpose. It will not be practicable for the military medical officer to carry with him in the field any large supply of the elegant pharmaceutical preparations which may be so useful in the permanent stations or in civilian work. The necessities of field work are of two kinds:

1. Portability of supplies.
2. Adaptability of the supply table.

To these may be added a third feature, represented in the person of the medical officer himself,—

3. The ability to utilise the therapeutic means afforded him.

It is not necessary to go into detail upon these points, but the ability to utilise military medical supplies is of much importance. In National Guard work, where an encampment is for but a short period, it will be possible as well for medical officers to take with them any additional agents which their own methods of practice cause them to feel to be needed. This plan, however, is to be discouraged because it violates the chief object of the annual encampment, which is to afford to the men, unobstructed by their ordinary avocations, the opportunities of military experience in a condition as nearly approaching actual service as may be. While the private soldier and the officer of the line are as far as possible adapting themselves to the military rules and regulations of the nation, it is desirable that the medical officer should not render himself conspicuous by departing from them. Strictly speaking then, he should take with him to camp nothing but the remedies designated by the nation for his work. He should make a careful study of the agents afforded in the field supply table, one of his chief occupations during the period of encampment, and if possible he should have taken up the subject carefully beforehand. He should be prepared to use the medicine furnished, as substitutes for other similar ones which he may have been accustomed to employ in his general practice. The writer realises how essential this is, from his experience with volunteer medical officers during the Spanish War, where many of them were greatly embarrassed by the lack of the good old standbys, which they had been administering in their civil practice but which were not available in the Army supply table, although agents equally efficient and effective were there offered in abundance.

*Military Medicine.*—Typhoid fever, general infectious diseases, diseases of the gastro-intestinal tract, diseases of dissipation and heat exhaustion are worth especial consideration. Although military medicine resembles civil practice in so many respects that, aside from the features of prophylaxis which will come under consideration in connection with military hygiene, but little need be said at this point.

*Military Surgery.*—In connection with military surgery especial attention should be given to the first aid dressing and to the various pouches and cases which are furnished the medical officer, in order that they may be readily utilised.

*Prophylaxis.*—Even more important than therapeusis is prophylaxis, a feature of medical work which has but little vogue in civil life, but which is of vital importance in army service. Here lies perhaps the most difficult duty of the National Guard surgeon. Accustomed in the work of civil life to wait for an invitation before extending the healing hand; wont to be bound down by the ethical requirement of not seeking or soliciting practice, it goes vastly against the grain of the civilian practitioner, recently or temporarily injected into military life, to publicly foist his sanitary opinions unasked or undesired upon his comrades or superiors.

But the conditions of military service, where large aggregations of men are accumulated with reciprocal action upon one another, are fraught with a myriad opportunities of infection and innumerable possibilities of disease. A single bit of neglect will open the door for a thousand misfortunes. With tuberculosis, typhoid, dysentery, typhus, smallpox, measles, mumps, yellow fever, malaria, tetanus, and a host of other affections crowding the sanitary picket line in search of an opening for their destructive action, it well behooves the medical man to be constantly on guard, and to continually keep before the service the dangers which he sees and, possibly, alone appreciates. The most important study then of the physician to whom the duties of military service may come is military hygiene and sanitation. This is almost of paramount importance and over and above everything else.

The food and water, the wastes and excreta, sanitary inspection and first aid, are all important topics to be taken up.

### II. MEDICO-MILITARY ADMINISTRATION.

The military portion of the work of the medical officer of the state forces is more of a novelty to the newly commissioned surgeon than the professional work of the specialty. He should remember that he is now a military man and should not fail to qualify himself to properly fill the new position. He should become familiar with his military duties, should learn his proper position in military maneuvers, should familiarise himself with the methods of handling the arms which he is entitled to carry, and should be in all respects a soldier as well as a doctor. The duties of a military character which are peculiar to him however, are themselves important and should be considered with the greatest care.

The examination of recruits, the definite organisation of work, with careful attention to its details, the recognition and appreciation of property accountability, are all matters which must be cultivated by the militia surgeon. He must learn to command men, not only those of his Hospital Corps, but the sick. The title of "Doc" or "Doctor" must be absolutely rejected, and he and his men must learn that the military salute and proper deference to rank is an essential feature of military service. In no place is his work more important than in connection with camp location and organisation, and in no place is there more need for insistence upon his rights and duties.

The proper organisation of the sanitary service is a matter of the highest importance to him. In some states such organisations are already in existence and need but a little improvement to bring them to the line of highest efficiency. In others organisation is defective or wanting and the medical officers, having learned what is necessary and desirable, should proceed to the correction of the defects and the filling of the vacua.

Paper work is a subject upon which a great deal of emphasis must be laid, experience having shown it perhaps to be the weakest feature of the medical officer's work. The value of records to a medical officer can be appreciated now by many who did not comprehend their use at the time, because of the numerous applications for pension which have been made by the sick who were under their charge. Among hundreds, and in some instances, thousands of cases which were treated in regimental and division hospitals, the recollection of a single instance is impossible without records, unless there should have been some exceedingly unusual or conspicuous incident to impress it upon the memory. The correct record not only protects the government from the assaults of unworthy claimants upon its treasury, but it assists in the obtaining of relief to worthy men who have suffered disabilities through military service. The proper keeping of the records then is a duty, not only to the government, which the medical officer has sworn to maintain and abide by, but an equal duty to the comrades who may have been entrusted to his care by friends at home or by the kindly concern of a generous nation.

Paper work then is of an importance of the first class and second only to therapeusis and prophylaxis. No National Guard medical officer should fail to make a thorough study of the various blanks and forms used for hospital purposes. Many of the states employ blanks of their own devising. The writer can hardly see the need, or even desirability, of such a condition. There are of course a number of blanks prescribed for use by the national service, with which the state troops must be fused in case of general hostilities, some of which are necessary in the national guard service, but those papers which are needed should be identified with those of the national government in order that the fusion, when it comes, may be attended with as little friction as possible. It is hardly possible that perfection should attend any piece of work, and so it can hardly be asserted that the forms and papers of the Army are perfect. Were such the case the changes which not infrequently occur would not be made. But the fact stands, nevertheless, that these are the forms and records which must be used when troops are mustered into the national service and with all their weaknesses and deficiences, it is probable that, having been devised by men of long experience in military affairs, they approximate very closely to the necessities of the service.

It is especially advised then that the state forces abandon the use of special forms for these records and utilise those of the national government. Were this done, and were the medical officers appreciative of the necessities of the case fully familiar with them, the need of especial instruction in public work upon the part of medical inspectors would be absent, and it would be practicable to devote far more time to professional work proper.

### III. THE EVOLUTION AND MAINTENANCE OF A MEDICO-MILITARY ESPRIT DE CORPS.

Enthusiasm is the mother of efficiency. The man who has the greatest interest in his work is the man who accomplishes the most for his cause. Those troops which are animated by the highest degree of patriotism are those which perform the greatest feats of arms. There are few officers of the military services, whether state or national, who are not animated by sincere patriotism. Second to this comes loyalty to one's state. Happily in this day national patriotism is recognised everywhere as paramount to state loyalty. Every medical officer should, nevertheless, be imbued with the deepest affection for the state which he is commissioned to serve, and should therefore be the more ready to disdain no labor nor exertion, physical or mental, which would add to his efficiency as one of its defenders. The observance of the points which have been all too inadequately presented in this paper is essential. The carrying out of the plan indicated will take time and some labor, but the result in efficiency and in loyalty will be beyond calculation.

No method of increasing one's efficiency and enthusiasm is greater than that of contact and attrition with men who are interested along similar lines. This has been recognised in some of our states by the organisation of medical officers' schools and medical officers' associations. The school for medical officers, maintained by the Volunteer Militia of Massachusetts, is of the highest value to that excellent service, while the Association of Military Surgeons of the State of Illinois cannot be underrated as a contributor to the efficiency of that superb medical corps. Organisations of this kind, whether schools or associations,—they mean the same whatever their name,—are of the highest value to the medical officer, and where they exist no man should fail to connect himself with them and participate actively in their work. Where they do not exist, efforts should not be lacking to bring them into existence.

But whatever the state relations may be along these lines, no medical officer should fail to avail himself of the incalculable advantage to be derived from membership in the Association of Military Surgeons of the United States. This organisation indeed began as an organisation of the medical officers of the National Guard, and its work has developed along lines of the highest character until it is now the greatest military medical association in the world. The opportunities of developing esprit de corps afforded by its meetings are inestimable. No man can mingle with the enthusiastic and interested participators in its conventions without developing added enthusiasm in his work. The monthly Journal, which reaches every member, whether he is or ever has been able to attend the meetings or not, brings regularly its cargo of information, original and selected, to the members of the Association. The prizes offered for the discussion of important military medical subjects have resulted in the production of numerous brochures of the greatest value to the profession. The activities of the Association have been mighty in gaining recognition for military medicine, and so long as this organisation lasts, military medicine cannot recur to the conditions which existed before its formation. It is then not only a privilege but a duty for every medical officer of the National

Guard, as well as for those of the national services, to identify himself with military medical progress and take interest in the great Association of Military Surgeons, which will reward his interest a thousand times by the encouragement, advantages and growth which it will contribute to his professional career.

### CONCLUSION.

In the preparation of this essay many points of an apparently elementary character have been brought out, mainly matters with which the Army medical officer becomes familiar by association with the service, but which the National Guard officer cannot learn from that source. Many military features, however, it has been impossible to bring out because of the limits of space, although it is realised that they are extremely essential. The writer was astonished during the Spanish-American War, when seated upon a hotel porch, by the side of the Corps commander, to see a volunteer Colonel pass in the street and salute the commanding General by holding his forage cap in the right hand, over his left shoulder, a little point which military experience upon the part of the Colonel, would have shown to be entirely incorrect, but which was not corrected by the commander because it was, as a matter of fact, a genuine evidence of the Colonel's desire to extend the highest military courtesy. There is no available book upon military etiquette or medico-military etiquette, a matter which the writer much regretted upon his own entrance into the service. For this reason he has admitted to this paper a number of points which might have been excluded were he writing for officers who have had regular army experience, or had there been an available textbook to which he might have referred the medical officer of the state forces for information.

To sum up the whole subject we may conclude then, as the consequence of our consideration of the training of the medical officer of the state forces, that to best qualify him for local service and for mobilisation with national troops,—

1. There is need of special study upon the part of the medical officers of the National Guard and state troops to fit themselves for work in their own forces, not only in local service, but in connection with the national services in time of war and in peaceful mobilisation.

2. The most essential feature in the effort to secure harmonious action with other state troops and with the national forces is uniformity of training, particularly along the lines of medico-military administration.

3. The most important element of the medical officer's training is confessedly the highest grade of efficiency in military hygiene, medicine and surgery. But of great importance and overlapping these to a considerable degree, is a familiarity with medico-military administration, without which, indeed, the proper practice of military medicine, surgery and hygiene will be impossible. The medical officer must become deeply acquainted with the proper methods of selecting recruits, he must be able to control his men, commanding the respect, not only of the Hospital Corps, but of the sick under his direction; he must be an authority beyond dispute upon camp location and organisation; he must understand the principles and practice of field hospital construction and composition; he must have experience in the instruction and drill of the sanitary soldiers; and he must be prepared to protect his service and assist his comrades by a thorough acquaintance with the records of his department.

4. To crown all these and to add to his efficiency in every respect, there must have developed in him an enthusiasm based upon loyalty to the nation and interest in his profession, which shall impel him at all times to labor unceasingly and incessantly for the good of the service which has honored him with its commission.

By pursuing then the lines of work and of thought, which have been altogether too inadequately suggested, the medical officer of the National Guard of the United States will find his department *semper paratus* for all the emergencies of peace within the limits of the commonwealth which he is serving, and ever ready, in case the dark clouds of national or international conflict should overshadow the horizon of the nation's glory, to at once unite and coalesce with the forces which the mother country has maintained as a nucleus about which all military operations shall center.

CARLISLE, PA.

